# Ferroptosis: A Double-Edged Sword in Gastrointestinal Disease

**DOI:** 10.3390/ijms222212403

**Published:** 2021-11-17

**Authors:** Chengfei Xu, Ziling Liu, Jiangwei Xiao

**Affiliations:** Department of Gastrointestinal Surgery, Chengdu Medical College, Chengdu 610500, China; cos119@163.com (C.X.); nsmc2007@163.com (Z.L.)

**Keywords:** ferroptosis, ischemia/reperfusion injury, inflammatory bowel disease, gastric cancer, colorectal cancer

## Abstract

Ferroptosis is a novel form of regulated cell death (RCD) that is typically accompanied by iron accumulation and lipid peroxidation. In contrast to apoptosis, autophagy, and necroptosis, ferroptosis has unique biological processes and pathophysiological characteristics. Since it was first proposed in 2012, ferroptosis has attracted attention worldwide. Ferroptosis is involved in the progression of multiple diseases and could be a novel therapeutic target in the future. Recently, tremendous progress has been made regarding ferroptosis and gastrointestinal diseases, including intestinal ischemia/reperfusion (I/R) injury, inflammatory bowel disease (IBD), gastric cancer (GC), and colorectal cancer (CRC). In this review, we summarize the recent progress on ferroptosis and its interaction with gastrointestinal diseases. Understanding the role of ferroptosis in gastrointestinal disease pathogenesis could provide novel therapeutic targets for clinical treatment.

## 1. Introduction

**Figure 1:**
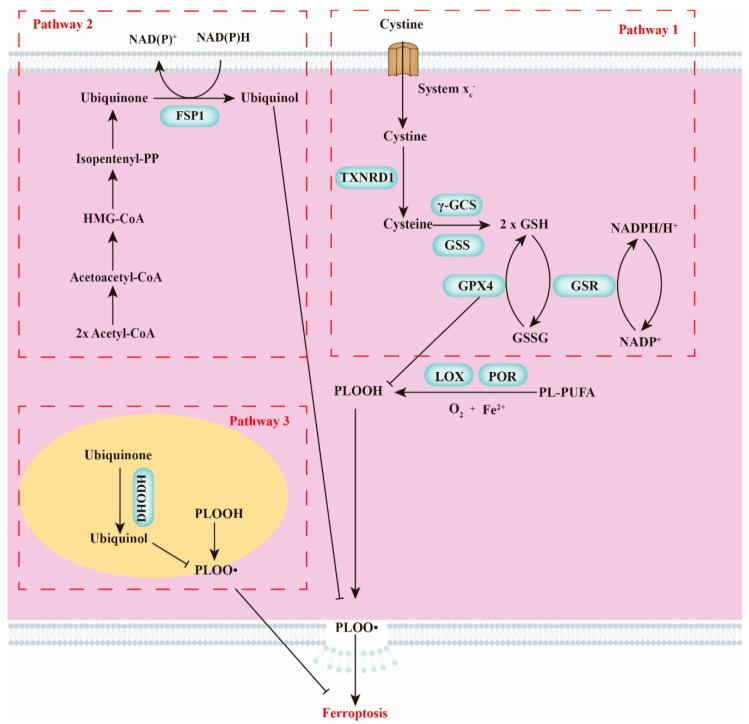
General view of ferroptosis. Footnote: Ferroptosis is triggered by the accumulation of oxidized and toxic phospholipids. This figure presents the three potential regulatory pathways of ferroptosis. TXNRD1, cysteine by thioredoxin reductase 1; γ-GCS, γ-Glutamylcysteine synthetase; GSS, glutathione synthetase; LOX, lipoxygenase; POR, P450 oxidoreductase; GSS, glutathione synthetase.

Since 2012, ferroptosis has been identified as a novel form of regulated cell death (RCD) that differs from necroptosis, apoptosis, and autophagy [1]. Ferroptosis is accompanied with accumulation of lipid reactive oxygen species (ROS) that derived from iron metabolism. The primary characteristics of ferroptosis are condensed mitochondria and increased bilayer membrane density [1,2]. Recently, numerous evidences indicate ferroptosis is regulated by a variety of signals from different organelles (such as, lysosomes, mitochondria, and the endoplasmic reticulum) [3]. Briefly, iron accumulation induces lipid peroxidation and subsequent trigger plasma membrane rupture [4]. Three regulatory pathways involved in the regulation of ferroptosis have been summarized in Figure 1. (1) The canonical pathway to regulate ferroptosis is the x_c_^–^–glutathione (GSH)-dependent pathway, which was first proposed in 2014 (pathway 1 in Figure 1) [5,6]. Phospholipid peroxidase glutathione peroxidase 4 (GPX4) is the only known GPX that is involved in the conversion of toxic lipid hydroperoxides (L-OOH) into nontoxic lipid alcohols (L-OH) under normal physiological conditions [7]. As a cofactor of GPX4, cellular GSH levels are regarded as a critical component that regulates ferroptosis [8]. Since System x_c_^–^ regulates the transportation of cystine (substrates to synthesize cysteine), System x_c_^–^ is proposed to have a direct relationship with ferroptosis [9]. (2) Ferroptosis suppressor protein 1 (FSP1)-dependent pathway was reported as a novel pathway to manipulate ferroptosis (pathway 2 in Figure 1) [10,11]. FSP1 is the predominant ferroptosis resistance factor in this pathway, which catalyzes the conversion of ubiquinone to ubiquinol and prevents lipid peroxidation in the cellular membrane. (3) The dihydroorotate dehydrogenase (DHODH)-dependent pathway was identified in 2021 (pathway 3 in Figure 1) [12]. DHODH mediates ferroptosis by reducing ubiquinone to ubiquinol, which specifically occurs in the mitochondria. Compelling evidence indicates that ferroptosis is involved in the regulation of multiple diseases in different organs, such as liver, lung, pancreas, and breast [13,14].

## 2. Crucial Modulators of Ferroptosis

### 2.1. GSH Homeostasis

Accumulated iron triggers ferroptosis by producing excessive ROS and inducing lipid peroxidation. Lipophilic anti-oxidants (such as Fer-1) could efficiently prevent the cell from ferroptosis [15]. As a critical component of cellular antioxidant defense system, glutathione (GSH) prevents the accumulation of ROS [16]. Its deficiency is associated with massive lipid peroxidation, dysregulated cellular functions and even cell death. GSH is synthesized from l-cysteine, l-glutamate, and glycine; therefore, cellular availability of these amino acids could directly affect the concentration of GSH. System Xc^−^ cystine/glutamate antiporter, composed of a heavy-chain subunit (CD98hc, SLC3A2) and a light-chain subunit (xCT, SLC7A11), mainly mediates the exchange of extracellular L-cystine and intracellular l-glutamate [17]. Erastin, a small molecular inhibitor of System Xc^−^, could deprive the cellular cysteine and impair the intracellular GSH homeostasis, leading to intracellular GSH deficiency and ferroptosis [9]. Similar to Erastin, sulfasalazine (SAS) could also block System Xc^−^ function and trigger ferroptosis in multiple cancer cell lines [18]. After converting cystine into cysteine by β-mercaptoethanol, cysteine could be transported into the cell bypass System Xc^−^ [19]. Thus, β-Mercaptoethanol treatment prevents cells from ferroptosis induced by System Xc^−^ inhibition [20].

As a master regulator of ferroptosis, phospholipid hydroperoxide glutathione peroxidase (GPX4) prevents the toxicity of lipid peroxides and protects the membrane lipid bilayers by converting toxic lipid hydroperoxides (L-OOH) into nontoxic lipid alcohols (L-OH) [21]. GPX4 is a critical selenoprotein, which is critical to prevent ferroptosis [22,23]. RSL3 is the other small molecule compound involved in the regulation of ferroptosis, which could directly target and inhibit GPX4 activity through covalent bonding [1]. GSH is the co-factor of GPX4, the deficiency of which could also indirectly block the function of GPX4 [8]. Thus, GSH depletion and/or GPX4 inhibition play an important role in the process of ferroptosis.

### 2.2. Polyunsaturated Fatty Acids

Accumulated ROS could directly react with polyunsaturated fatty acids (PUFAs) of plasma membranes and trigger ferroptosis. Recent advances indicate that plasma fatty acid have intimate relationship with ferroptosis. Fatty acids (FAs) are classified into saturated fatty acids (SFAs), monounsaturated fatty acids (MUFAs), and PUFAs depending on the degree of hydrocarbon chain saturation of fatty acid. As direct targets of intracellular ROS, PUFAs can be oxidized into lipid peroxides [24]. It is now clear that ferroptosis mainly occurs in the plasma membrane phosphatidylethanolamines (PEs), which consist of two specific fatty acid chains—arachidonic acid (AA) and adrenic acid (AdA) [25]. Blockage of fatty acid elongation by very-long-chain fatty acid protein 5 (ELOVL5) and fatty acid desaturase 1 (FADS1), which are involved in AA and AdA synthesis, could efficiently inhibit ferroptosis [26]. Acyl-CoA synthetase long-chain family member 4 (ACSL4) is a critical lipid metabolism regulator that is involved in the regulation of ferroptosis [27]. This might attribute to the fact that ACSL4 specifically drives esterification of AA and AdA into PE. When replacing PUFAs with MUFAs in the plasma membrane, cells could enter into a ferroptosis-insensitive state [28]. Even though lysophosphatidylcholine acyltransferase 3 (LPCAT3) also found to catalyze the insertion of acylated AA into membrane phospholipids and trigger ferroptosis [29], whereas its effect on ferroptosis was mild compared with ASCL4 [30]. It is worth noting that replacing PUFAs with MUFAs in the plasma membrane could hinder lipid ROS accumulation and prevent the ferroptosis [28].

### 2.3. Cellular Iron Pool

Iron is a redox-active reagent, which manipulates ROS production via Fenton reaction and induces lipid peroxidation [31]. Free cellular iron is essential for the progress of ferroptosis, as it serves as a cofactor for lipid-oxidizing lipoxygenases (LOXs), which is a central player in ferroptosis. Iron could react with PLOOH and produce the free radicals like, PLO• and PLOO• [32]. Iron chelators could largely diminish lipid peroxides and prevent the cell from ferroptosis [33]. Compelling evidence indicate that genes involved in iron metabolism are indispensable elements in ferroptosis.

Intracellular iron levels are mainly determined by the processes of iron storage and export. Iron-regulatory proteins 1 and 2 (IRP-1 and IRP-2) are critical transcriptional factors that participate in iron metabolism, which are manipulated by intracellular compart iron level [34]. For instance, both transferrin (TF) and transferrin receptor 1 (TFR1), which regulate the transportation of iron from the extracellular into the cell, are potential targets of IRP1 and IRP2 [35]. Conversely, ferroportin (also known as solute carrier family 40 member 1 (SLC40A1) mainly regulate the iron export, which is considered as the negative regulator of ferroptosis [36]. In addition to regulating cellular iron level though iron transport system, free cellular iron could be also regulated by iron storage. Ferritin is the other target of IRP1 and IRP2, that binds to the free cellular iron and prevent the cell from ferroptosis [37]. Manipulating cellular iron level either through reducing iron storage or increasing iron uptake could induce iron overload and lead to ferroptosis.

## 3. The Role of Ferroptosis in Gastrointestinal Disease

**Figure 2:**
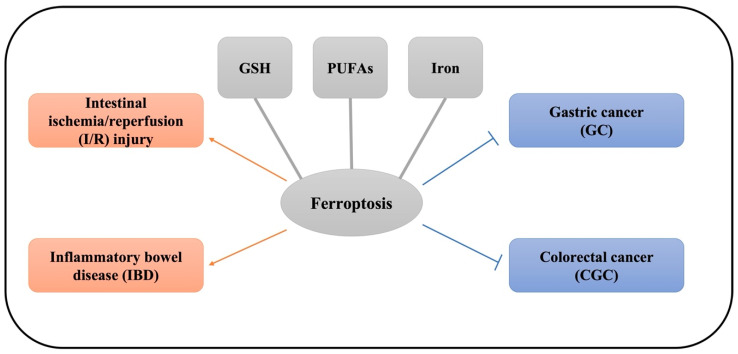
The role of ferroptosis in gastrointestinal disease. Footnote: Ferroptosis is a double-edged sword in gastrointestinal disease, which is mainly regulated by GSH homeostasis, plasma PUFA concentration, and liable iron pool. On one hand, ferroptosis activationhas a negative impact on intestinal I/R injury and inflammatory bowel disease (IBD). On the other hand, ferroptosis activation could trigger cell death and alleviate gastric cancer (GC) and colorectal cancer (CGC). Red arrows indicate ferroptosis activation induce the corresponding diseases. Blue arrows indicate ferroptosis activation alleviate the corresponding diseases.

The gut has received great attention for a long time. It does not simply act as a digestive system for nutrient absorption, but also perform as a barrier to exclude antigens and pathogens from external environment. Gastrointestinal disease directly leads to the morbidity and mortality worldwide each year. Understanding the molecular pathology of the gastrointestinal tract may help identify novel therapeutic targets for gut disease. Cell death has been found as a critical therapeutic target for gastrointestinal diseases. Previously, necroptosis, apoptosis, and autophagy have been demonstrated to be intimately involved in the regulation of gastrointestinal disease [38]. Recently, compelling evidence has indicated that ferroptosis also largely participates in the manipulation of gut health. In this review, we summarized recent progress among ferroptosis with intestinal I/R injury, inflammatory bowel disease (IBD), GC, and CRC (Figure 2). Crucial molecular players and potential therapeutic targets involved in regulation of gastrointestinal disease is summarized in Table 1.

### 3.1. Intestinal I/R

As a challenging clinical intestinal disease with high morbidity and mortality rates, intestinal I/R injury occurs in response to multiple pathological conditions, such as hemorrhagic shock, traumatic shock, strangulated intestinal obstruction, severe burns, and chronic and acute mesenteric ischemia [47,48]. Previously, intestinal I/R injury was reported to have an intimate relationship with RCD, such as apoptosis [49] and necroptosis [50]. Recently, the connection between intestinal I/R injury and ferroptosis has also been identified. Attenuated GSH, accumulated ROS and subsequently increased lipid peroxidation that accompanied by intestinal I/R injury have been found to trigger ferroptosis [51,52]. Under ischemic conditions, the progression of ferroptosis is enhanced by downregulation of negative regulators (GPX4 and ferritin heavy chain 1 (FTH1)) and upregulation of ACSL4, a positive regulator of ferroptosis [39]. Specifically, special protein 1 (Sp1) directly binds to the ACSL4 promoter region and upregulates the expression of ACSL4. Pharmacologically, inhibition of ASCL4 protected intestinal against I/R induced lipid peroxidation, and alleviated subsequent intestinal damage [30,39]. Importantly, liproxstatin-1, a ferroptosis inhibitor, has been demonstrated to ameliorate I/R-induced intestinal injury [39]. In addition, iron chelators deferoxamine (DFO) also prevents the intestinal I/R-induced lipid peroxidation [53].

Intriguingly, recent findings also indicate that intestinal I/R injury-induced ferroptosis is partially regulated by modification of the gut microbiota and metabolites [54]. Capsiate (CAT) is an intestinal microbiota-generated metabolite that is significantly attenuated in response to intestinal I/R injury. CAT activates transient receptor potential cation channel subfamily V member 1 (TRPV1), which further upregulates the expression of GPX4 and protects the cell from ferroptosis. Collectively, I/R-induced intestinal injury could be caused by ferroptosis, but more research is needed to clarify the underlying mechanisms. Overall, ferroptosis tends to have a negative impact on intestinal I/R injury. Inhibition of ferroptosis could be targeted therapeutically to relieve the intestinal I/R. More studies are still required to decipher the connection between intestinal I/R injury and ferroptosis in the future.

### 3.2. The Role of Ferroptosis in IBD

Inflammatory bowel disease is a chronic inflammatory disorder of the gastrointestinal tract that includes ulcerative colitis (UC) and Crohn’s disease (CD) [55]. Elevated levels of ROS and malondialdehyde (MDA) in colitis provide suggestive evidence for the presence of ferroptosis in these diseases [56]. Ferroptosis-related genes are significantly changed in UC patients and dextran sulfate sodium (DSS)-induced murine colitis. Intestinal epithelial cells isolated from UC patients and mice with colitis exhibited increased prostaglandin H synthase 2 (PTGS2) and decreased GPX4, which are generally considered biomarkers of ferroptosis [40]. Similarly, Chen [41] reported that DSS-induced UC in mice increased the expression of cyclooxygenase-2 (COX2) and ACSL4 while decreasing the expression of GPX4 and FTH1. Consistent with UC, GPX4 activity is also reduced in intestinal epithelial cells from patients with CD [57,58]. Inhibition of ferroptosis by ferrostatin-1 (Fer1) alleviated colitis induced by DSS challenge [40].

To date, two potential pathways have been proposed for ferroptosis to regulate UC. First, ferroptosis regulates UC through ER stress-mediated intestinal epithelial cell death [40]. Mechanistically, phosphorylated NF-κBp65 interacts with eIF2α and inhibits ER stress-mediated ferroptosis of intestinal epithelial cells, indicating that NF-κBp65 might represent a potential therapeutic target for UC [40]. Furthermore, the Nrf2/HO-1 pathway inhibits the NF-κB pathway, which subsequently suppresses secretion of the proinflammatory cytokines interleukin-1β (IL-1β), interleukin-6 (IL-6), and tumor necrosis factor alpha (TNF-α) [59]. Some preliminary evidence indicates that ferroptosis regulates DSS-induced UC through the Nrf2/HO-1 signaling pathway [41]. All these findings indicate that ferroptosis seems to exacerbate inflammatory bowel disease. Thus, therapeutically blocking ferroptosis signaling could be an efficient way to alleviate chronic inflammatory disorder.

### 3.3. The Role of Ferroptosis in Gastric Cancer (GC)

GC is the fourth most common cancer worldwide and the second leading cause of cancer-related death after lung cancer [60]. There are no specific symptoms for the early diagnosis of GC; therefore, most patients are diagnosed at advanced disease stages. Identification of GC during early stages is critical for increasing the five-year survival rate of GC [61]. Multiple genes related to ferroptosis could be used as diagnostic and prognostic markers in GC patients (Table 2). Some ferroptosis related genes directly regulate GPX4/GSH system. For example, MYB acts as a proto-oncogenic transcription factor, which regulates the expression of GPX4 during ferroptosis [62]. Phosphoserine aminotransferases 1 (PSAT1) could suppress ferroptosis by stimulating GSH synthesis [63]. The Gln importer SLC1A5 is required for Gln uptake, which is a critical substrate for GSH synthesis [64]. In addition, nuclear protein 1 (NUPR1) and TF manipulate ferroptosis via the modification of liable iron. NUPR1 is a transcriptional regulator to regulate the expression of lipocalin 2 (LCN2), which could alleviate iron accumulation and lead to ferroptosis resistance [65]. TF is an iron-binding protein, which modulates the transportation of iron via TfR1-mediated endocytosis [66]. Furthermore, genes that regulate the production of ROS and lipid peroxides are also identified as biomarker for ferroptosis, such as LOX, NADPH oxidase 4 (NOX4), and ZFP36. The LOX family of enzymes is iron-dependent, the inhibition of which decreases accumulation of lipid peroxides and subsequently blocks ferroptosis [67]. Increased NOX4 impairs mitochondrial function, induces lipid peroxidation, and promotes ferroptosis [68]. The RNA-binding protein ZFP36 is reported to regulate the cellular response to lipid peroxidation and protect cells from ferroptosis [69]. Intriguingly, ZFP36 seems to be a reliable gene for clinical prognostic prediction in GC, which was included by all four independent studies. Although a large discrepancy in ferroptosis-related genes was reported in these studies, the intimate relationship between GC and ferroptosis cannot be neglected. To date, it is still unclear the improvement extent for the early diagnosis of GC after using these ferroptosis-related genes.

GC has lower expression of GPX4, making it more susceptible to ferroptosis than normal intestinal cells [43]. Recent studies indicate that further inhibiting the activity of GPX4 efficiently induce the ferroptosis in GC. Apatinib is a small-molecule inhibitor of vascular endothelial growth factor receptor-2 (VEGFR2) that has been proven to be effective for the treatment of GC [74]. Interesting, it has been demonstrated that apatinib decreases the expression of GPX4 and inhibits the transcription factor sterol regulatory element-binding protein-1a (SREBP-1a) in GC cells [43]. The other therapeutic target to regulates ferroptosis in GC is the glutamate/cystine antiporter SLC7A11/xCT. As a classic ferroptosis inducer, erastin triggers ferroptosis and suppresses the survival of GC cells through inhibition of the SLC7A11/xCT system [42].

In addition to the GPX4/GSH system, multiple functional proteins that directly involved in the regulation of ferroptosis also changed significantly in GC. For example, stearoyl-CoA desaturase 1 (SCD1) participates in the conversion of saturated fatty acids into MUFAs [75]. High expression of SCD1 is associated with the growth of GC cells and the suppression of ferroptosis, which could be a prognostic biomarker for predicting GC [44]. ELOVL5 and FADS1 participate in the regulation of n-PUFA biosynthesis (AA and AdA synthesis) [26]. In mesenchymal-type GC cells, ELOVL5 and FADS1 expression is upregulated [26], which indicates that GC cells are accumulated with PUFA and are more sensitive to ferroptosis. It worth noting that, ferroptosis is also indirectly regulated by transcription factors. Cytoplasmic polyadenylation element binding protein (CPEB) is a critical factor that regulates mRNA translation [76]. Low expression of CPEB has been found to be connected to the metastasis of GC [77]. Mechanistically, decreased expression of CPEB1 increases the expression of twist1, an inhibitor of activating transcription factor 4 (ATF4), thereby attenuating the ATF4/ChaC glutathione specific gamma-glutamylcyclotransferase 1 (CHAC1) pathway [77]. Decreased CHAC1 is proposed to inhibit GSH degradation and further protect GC cells from ferroptosis [78].

Recently, more evidence has indicated that miRNA and circular RNA dysregulation is also related to ferroptosis-related GC. For example, physcion 8-O-β-glucopyranoside (PG) has been found to promote ferroptosis by increasing GLS2 expression through miR-103a-3p in GC [79]. Phosphate-activated glutaminase (GLS2) is a p53-inducible regulator of glutamine metabolism that converts glutamine to glutamate for GSH synthesis [80]. Thus, miR-103a-3p regulates GC through the alteration of cellular GSH levels. In addition, levobupivacaine, an amide-based local anesthetic, is reported to inhibit GC through the upregulation of miR-489-3p, which targets SLC7A11/xCT [81]. Furthermore, circ_0008035 was reported to be upregulated in GC tissues and cells and to act as a sponge for miR-599 [82]. miR-599 further target eukaryotic initiation factor 4A1 (EIF4A1) and regulate ferroptosis through an unknown mechanism [82]. Intriguingly, miRNAs and circular RNAs can be transported by exosomes and regulate ferroptosis through paracrine signaling in the tumor microenvironment. Some preliminary evidence indicates that the TME inhibits ferroptosis and promotes the growth of GC cells through exosomes [83]. Specifically, exosomal miR-522 secreted from cancer-associated fibroblasts (CAFs) targets arachidonate lipoxygenase 15 (ALOX15), which is a lipid peroxidation enzyme [83].

*Helicobacter pylori* induced GC could also be alleviated by the activation of ferroptosis. Globally, more than 50% of people are infected with *Helicobacter pylori* [84]. The most crucial cause of sporadic distal GC is *Helicobacter pylori* infection [85]. Previously, miR-375 was reported to inhibit *Helicobacter pylori*-induced GC by decreasing Janus kinase 2-signal transducers and activators of transcription (JAK2-STAT3) signaling [86]. A recent study found that miR-375 also directly targets the glutamate/cystine antiporter SLC7A11/xCT, which decreases cellular glutathione levels and triggers the process of ferroptosis [87].

Collectively, ferroptosis might be involved in the regulation of GC. Targeting ferroptosis may be a promising method for the treatment of GC in the future. However, novel components are in need to be identified in order to look for therapeutic targets that can efficiently trigger ferroptosis and alleviate GC.

### 3.4. The Role of Ferroptosis in Colorectal Cancer (CRC)

As the second most lethal cancer and the third most prevalent malignant tumor in the world, CRC caused 0.94 million cancer-related deaths in 2020 [88,89]. Currently, surgery, chemotherapy, and targeted therapy have been widely used for CRC treatment, whereas some patients still exhibit resistance to these therapies [90]. A recent study confirmed that ferroptosis might be a substitute strategy for killing chemotherapy-resistant CRC cells [70]. In addition, mounting evidence suggests that ferroptosis-related genes could be used as biomarkers to predict CRC (Table 3). Even though some prognostic signatures in CRC are consistent with those in GC, such as NOX4, most of the genes that available for the prediction of CRC and GC are different. Genes used to build a prognostic model for the prediction of CRC have intimate relationship with ferroptosis. Some ferroptosis related genes focuses on the regulation of GPX4/GSH system. For example, activating transcription factor 3 (ATF3) promotes ferroptosis by inhibiting amino acid antiporter System Xc^−^ [91]. Ribonucleotide reductase subunit M2 (RRM2) can maintain cellular GSH concentration and perform an anti-ferroptotic effect [92]. Cyclin-dependent kinase inhibitor 2A (CDKN2A) sensitizes cells to ferroptosis via downregulating the expression of SLC7A11 [93]. Growth-differentiation factor 15 (GDF15) deficiency leads to the downregulation of SLC7A11 and promotes ferroptosis [94]. In addition, genes involved in iron pool regulation also act as powerful prognostic indicators for CRC. Accumulation of mitochondrial labile iron is associated with enhanced expression of the tumor suppressor thioredoxin-interacting protein (TXNIP) [95]. Cysteine desulfurase (NFS1) activity is important for maintaining the iron–sulfur co-factors, the suppressing of which could predispose cells to ferroptosis [96]. Recently, ferroptosis-related lncRNAs have also been available for the prediction of colon cancer [97]. Circular RNA also seems to play a crucial role in ferroptosis. A study showed that circABCB10 acts as a sponge of miR-326 to regulate CCL5 expression, which ultimately regulates ferroptosis in CRC [98]. Although the candidate genes identified in different studies are largely inconsistent, these studies provide some preliminary evidence that ferroptosis is involved in the progression of CRC.

Targeting GPX4/GSH system is an efficient way to inhibit the growth of CRC cells. In CRC stem cells, the expression of SLC7A11 is extremely high; thus the cellular GSH is high and ROS level is low compared to parental levels [103]. Erastin is an inhibitor of SLC7A11, which could induce ferroptosis and attenuate the progression of CRC cancer stem cells [103]. Similarly, Talaroconvolutin A (TalaA), a new ferroptosis inducer, also inhibits the proliferation of CRC cells by decreasing SLC7A11 and increasing arachidonate lipoxygenase 3 (ALOXE3) [104]. In addition, the benzopyran derivative 2-imino-6-methoxy-2H-chromene-3-carbothioamide (IMCA) prevents the growth of CRC cells by downregulating SLC7A11 through AMP-activated protein kinase (AMPK)/the mechanistic target of rapamycin complex I (mTORC1) signaling [105]. GPX4 is another critical target to regulate ferroptosis. RAS-selective lethal 3 (RSL3) is a specific small-molecule compound, which could directly bind with GPX4 and inhibit its activity. RSL3 treatment increases the accumulation of ROS and cellular labile iron in CRC cells, and finally triggers ferroptosis [106]. Conversely, Lipocalin 2, a critical protein to regulate iron homeostasis, could suppress ferroptosis by stimulating the expression of GPX4 and xCT [107].

Currently, the canonical tumor suppressor p53 and heme oxygenase-1 (HO-1) have been identified as two critical regulators involved in ferroptosis-related CRC. p53 seems to regulate ferroptosis through transcriptional or posttranslational mechanisms in colon cancer cells. A novel antitumor compound optimized from the natural saponin albzia bioside A was demonstrated to trigger ferroptosis through the activation of p53 [108]. Mechanistically, p53 induces ferroptosis through inhibition of SLC7A11 or dipeptidyl-peptidase-4 (DPP) [45]. Cytoglobin (CYGB) is a cellular ROS regulator that regulates oxygen homeostasis and acts as a tumor suppressor [109]. Recently, CYGB was reported to regulate ferroptosis through p53-(Yes-associated protein 1) YAP1 signaling in CRC cells [110]. HO-1 increases available cellular iron, which can trigger ferroptosis [46]. Bioactive compounds isolated from *Betula etnensis* Raf. (Birch Etna) induce ferroptosis by upregulating heme oxygenase-1 (HO-1) expression in a human colon cancer cell line [111]. Similarly, Tagitinin C isolated from *Tithonia diversifolia* promotes ferroptosis through activation of Nrf2/HO-1 signaling in CRC cells [46].

Overall, similar to GC cells, CRC cells also seem to be sensitive to ferroptosis. Therapeutic effects could be achieved by inducing ferroptosis in these cancer cells. It is worth noting that although ferroptosis is related to CRC, but more research is needed to understand its underlying mechanisms.

## 4. Effects of Dietary Nutrients and Phytochemicals on Ferroptosis and Gastric Diseases

Numerous studies have demonstrated that eating habits are closely related to gastrointestinal disease. Recent advances also indicate that nutrient signals can regulate ferroptosis. First, iron enhances lipid peroxidation (converting PLOOH to the free radicals PLO• and PLOO•) through activation of LOX enzymes [32]. Increasing iron levels in the diet could directly increase cellular iron concentrations and the possibility of having UC [112,113]. Iron overload aggravated intestinal inflammation induced by DSS in rat model [114] In contrast, iron chelators could efficiently inhibit ROS generation and alleviate symptoms in IBD [115,116]. These evidence indicate an intimate relationship between ferroptosis and IBD. In addition, dietary iron decreased the expression of SLC7A11 and GPX4 through nuclear factor erythroid 2-related factor 2 (NRF2) and enhanced resistance to ferroptosis in CRC cells [117]. Thus, a low-iron diet could be a therapeutic method for the treatment of CRC. Second, selenium is a critical component of GPX4 [23]. As mentioned above, GPX4 is the only known selenoprotein that protects cells from ferroptosis. Dietary supplementation with selenium may enhance the activity of GPX4 and protect cells from ferroptosis and its related intestinal diseases in selenium-deficient individuals [118,119]. Third, ubiquinone (CoQ_10_) is a lipid-soluble antioxidant that regulates mitochondrial membrane potential and ROS generation [120,121]. To date, CoQ_10_ is pharmacologically used as an antioxidant supplement and to alleviate disorders related to mitochondrial dysfunction [122]. FSP1 reduces ubiquinone to ubiquinol and prevents lipid peroxidation [10,11]. However, whether dietary supplementation with CoQ_10_ could protect cells from ferroptosis is uncertain and require more study [123,124]. Fourth, dietary supplementation with PUFAs is an efficient way to promote ferroptosis, while MUFAs make cells more resistant to ferroptosis [28]. Arachidonic acid triggers IBD by inducing ferroptosis in *Gpx4*-deficient mice [57]. A westernized diet plays a major role in IBD onset and progression by modulating the intestinal bacteria [125,126]. Recently, the prevalence of IBD has rapidly increased not only in western countries but also in newly industrialized countries in Asia, the Middle East, Africa, and South America [127]. A westernized diet usually contains high levels of ω-6 PUFAs and AA, which indicates that these factors might induce IBD with the activation of ferroptosis. In addition, western foods contribute to a higher rate of CRC [128]. A recent study indicated that food consumption-induced CRC is also related to ferroptosis. Dietary administration of n-3 long-chain PUFAs and highly fermentable fiber increased the accumulation of cellular lipid ROS to induce ferroptosis and reduce CRC risk [129]. The role of ferroptosis is different among intestinal IR injury, IBD, GC, and CRC. To date, there is still limited evidence to support the prophylactic or therapeutic effects of dietary nutrients on ferroptosis related diseases. Current studies propose a hypothesis that a more balanced diet (balanced iron, selenium, and FA in diet) might be a better choice for maintaining gastrointestinal health.

In addition to dietary nutrients, compelling evidence indicates that phytochemicals also participated in the regulation of ferroptosis and relieves the symptoms of GC. For example, Jiyuan oridonin A (JDA) is isolated from the Chinese medicinal herb *Rabdosia rubescens* and has antitumor properties. A2, a novel derivative of JDA, has been found to decrease the expression of GPX4 and induce ferroptosis, ultimately decreasing the growth of GC cells [130]. *Actinidia chinensis* Planch. contains two antitumor components, ursolic acid and oleanolic acid, which promote ferroptosis by inhibiting GPX4 and SLC7A11/xCT proteins in GC cells [131]. As an active component of the Chinese herb *Salvia miltiorrhiza* Bunge (Danshen), tanshinone IIA (Tan IIA) has been found to have an antitumor effect in GC [132,133]. Mechanistically, Tan IIA increased lipid peroxidation and induced ferroptosis in GC by increasing p53 expression and decreasing xCT expression [134].

Recently, multiple bioactive components isolated from plants have also been identified to prevent CRC and induce ferroptosis [135,136]. It is worth noting that many compounds that regulate ferroptosis-related CRC are GPX4- and/or xCT-dependent. For example, honokiol, a biphenolic compound originally extracted from *Magnolia grandiflora* that has been reported to exert anticancer effects through the inhibition of GPX4 and activation of ferroptosis in CRC cells [137]. Besides, gallic acid, Chinese matrine, and Chinese herb *Curcumae rhizoma* have also been shown to induce ferroptosis in CRC cells by inhibiting GPX4 and SCL7A11 [138,139,140]. However, the underlying mechanism by which these bioactive components regulate the expression of GPX4 and SLC7A11 is still largely unknown and require further study. In addition to the GPX4-xCT system, certain active components also regulate CRC through other ferroptosis-related genes. For instance, bromelain, a mixture of proteases derived from pineapple stems (*Ananas comosus* L., family Bromeliaceae), downregulates ACSL4 through an unknown mechanism [141].

## 5. Conclusions and Perspectives

As a novel type of regulated cell death, ferroptosis is characterized by the accumulation of cellular iron and toxic phospholipids. Ferroptosis is widely implicated in the development of gastrointestinal diseases. Intriguingly, ferroptosis seems to be a double-edged sword for gastrointestinal disease. Inhibition of ferroptosis relieves the symptoms of intestinal I/R injury and IBD. However, the induction of ferroptosis using pharmacological agonists or bioactive compounds inhibits the proliferation of GC and CRC. This evidence indicates that the role of ferroptosis is different in gastrointestinal diseases. To date, most research indicates that ferroptosis in intestinal and GC cells is primarily regulated through targeting GPX4 or System X_C_^−^, which might partially be how this classical pathway was first identified. However, whether FSP1 and DHODH could be two other critical targets for regulating ferroptosis-related gastrointestinal disease is still unclear and requires further research. In addition, whether crosstalk between ferroptosis and other forms of regulated cell death occur in gastrointestinal diseases also remains to be clarified. Recent studies have proposed some ferroptosis-related genes as biomarkers for predicting CC and CRC. However, the candidate genes identified in different studies are largely inconsistent. It would be interesting if there were ferroptosis biomarkers that could indicate gastrointestinal disease severity. Collectively, ferroptosis signaling is a promising therapeutic target for the treatment of gastrointestinal disease and requires further investigation.

## Figures and Tables

**Table 1:** Crucial molecular players and potential therapeutic targets involved in regulation of gastrointestinal disease.

Gut Diseases	Genes	Compunds	Mechanism	References
Intestinal I/R	*GPX4, ASCL4*	Lip-1	Inhibit ferroptosis and ameliorate intestinal I/R	[30,39]
Inflammatory bowel disease	*GPX4, ASCL4, FTH1, NF-κBp65*	Lip-1, Fer-1, DFP, DFO	Inhibit ferroptosis and ameliorate inflammatory bowel disease	[40,41]
Gastric cancer	*GPX4, SLC7A11, SCD1, ELOVL5* and *FADS1*	Apatinib, Erastin	Induce ferroptosis and inhibit gastric cancer	[26,42,43,44]
Colorectal cancer	*GPX4, SLC7A11, p53, HO-1*	Erastin, Cisplatin, Bromelain	Induce ferroptosis and inhibit colorectal cancer	[45,46]

**Table 2:** Ferroptosis related genes used as diagnostic and prognostic markers in GC patients.

Ferroptosis Related Genes	References
*GABARAPL1, ZFP36, DUSP1, TXNIP, NNMT, MYB, PSAT1*, and *CXCL2*	[70]
*AKAP12, DUSP1, EFNA3, LOX, PIM1, SERPINE1, STC1*, and *ZFP36*	[71]
*NOX4, NOX5, SLC1A5, GLS2, MYB, TGFBR1, NF2, ZFP36, DUSP1, SLC1A4*, and *SP1*	[72]
*TCFBR1, MYB, NFE2L2, ZFP36, TF, SLC1A5, NF2,* and *NOX4*	[73]

**Table 3:** Ferroptosis related genes used as diagnostic and prognostic markers in CRC patients.

Ferroptosis Related Genes	References
*SLC2A3*, *ATF3*, *VLDLR*, *TXNIP*, *ZFP69B*, *ABCC1*, *NFS1*, *RRM2*, and *BID*	[99]
*TFAP2C, SLC39A8, NOS2, HAMP, GDF15, FDFT1, CDKN2A, ALOX12, AKR1C1, ATP6V1G2*	[100]
*ATP6V1G2, DRD4, JDP2, SLC2A3* and *VEGFA, ATG7, DUOX1, NOX4* and *PGD*	[101]
*AKR1C1, ALOX12, FDFT1, ATP5MC3,* and *CARS1*	[102]
lncRNAs (*LINC01503, AC004687.1, AC010973.2, AP001189.3, ARRDC1-AS1, OIP5-AS1*, and *NCK1-DT*)	[97]

## Data Availability

Not applicable.
